# An Automated Approach for Finding Spatio-Temporal Patterns of Seasonal Influenza in the United States: Algorithm Validation Study

**DOI:** 10.2196/12842

**Published:** 2020-09-04

**Authors:** Prathyush Sambaturu, Parantapa Bhattacharya, Jiangzhuo Chen, Bryan Lewis, Madhav Marathe, Srinivasan Venkatramanan, Anil Vullikanti

**Affiliations:** 1 University of Virginia Charlottesville, VA United States

**Keywords:** epidemic data analysis, summarization, spatio-temporal patterns, transactional data mining

## Abstract

**Background:**

Agencies such as the Centers for Disease Control and Prevention (CDC) currently release influenza-like illness incidence data, along with descriptive summaries of simple spatio-temporal patterns and trends. However, public health researchers, government agencies, as well as the general public, are often interested in deeper patterns and insights into how the disease is spreading, with additional context. Analysis by domain experts is needed for deriving such insights from incidence data.

**Objective:**

Our goal was to develop an automated approach for finding interesting spatio-temporal patterns in the spread of a disease over a large region, such as regions which have specific characteristics (eg, high incidence in a particular week, those which showed a sudden change in incidence) or regions which have significantly different incidence compared to earlier seasons.

**Methods:**

We developed techniques from the area of transactional data mining for characterizing and finding interesting spatio-temporal patterns in disease spread in an automated manner. A key part of our approach involved using the principle of minimum description length for representing a given target set in terms of combinations of attributes (referred to as clauses); we considered both positive and negative clauses, relaxed descriptions which approximately represent the set, and used integer programming to find such descriptions. Finally, we designed an automated approach, which examines a large space of sets corresponding to different spatio-temporal patterns, and ranks them based on the ratio of their size to their description length (referred to as the compression ratio).

**Results:**

We applied our methods using minimum description length to find spatio-temporal patterns in the spread of seasonal influenza in the United States using state level influenza-like illness activity indicator data from the CDC. We observed that the compression ratios were over 2.5 for 50% of the chosen sets, when approximate descriptions and negative clauses were allowed. Sets with high compression ratios (eg, over 2.5) corresponded to interesting patterns in the spatio-temporal dynamics of influenza-like illness. Our approach also outperformed description by solution in terms of the compression ratio.

**Conclusions:**

Our approach, which is an unsupervised machine learning method, can provide new insights into patterns and trends in the disease spread in an automated manner. Our results show that the description complexity is an effective approach for characterizing sets of interest, which can be easily extended to other diseases and regions beyond influenza in the US. Our approach can also be easily adapted for automated generation of narratives.

## Introduction

Large-scale spatio-temporal analyses and forecasts are becoming increasingly common for several diseases, such as influenza [1-4]. There is a lot of public interest in analysis of spatio-temporal trends relating to how these diseases are spreading across the United States—this includes statements about whether the season has officially started, a listing of regions which have differing levels of activity, and the contrast between the current season and earlier seasons. Such analyses have a broad readership and are popular among news media, the general public, and government agencies, as well as public health organizations; this is evidenced by spatio-temporal pattern reports [5,6] about the spread of influenza from news agencies and blogs.

Such patterns are typically identified manually by domain experts who have significant expertise on specific diseases. Data for such analyses often comes from public health agencies, such as the Centers for Disease Control and Prevention (CDC) [7] and World Health Organization. Reports generated by the CDC contain raw surveillance data on metrics (eg, activity level from outpatient visits and rates of hospitalization) across states in the US. In addition, summaries of regions with specific characteristics (eg, those which have high activity levels) are also included in the reports [7,8]. For instance, one CDC report [8] summarizes the states with high influenza-like illness activity for the week ending on March 4, 2017 with the number of states followed by a list of the state names.

Such descriptive listings are easy to construct from raw data but are tedious to read and do not provide deeper insight into the disease spread. In contrast, the analysis by Mashable [6] is a succinct description of the set of states which have widespread activity, namely, all states in the contiguous US, except Oregon. An analysis by the New York Times [5] was also a good and succinct description of the set of states which have reported widespread activity for 3 consecutive weeks. In addition to descriptions of the set of states with a particular activity level, sets exhibiting specific temporal patterns might also be of interest. An example is the set of states which maintained stable high activity for 3 consecutive weeks, ending in the week of January 27, 2018; most states had high influenza-like illness activity level 4 weeks prior, plus the states of New Jersey, New Mexico, Virginia, Washington, and Wyoming. Such descriptions involve identification of features common to these states, which provide additional insights on the outbreak.

The overall objective of our work was to automate the process of identifying interesting spatio-temporal patterns from disease surveillance data and generating succinct descriptions for them. In order to do this, we encoded the incidence data as binary matrices (presence or absence of a feature) and used techniques from pattern mining [9,10] in transactional data to find insights into epidemic spread; we demonstrated its utility using seasonal influenza in the US as a case study.

## Methods

### Data

We used the state level influenza-like illness activity indicator data available from the CDC [11]. In the data set, each state for each week during a given influenza season is assigned an activity level from 1 to 10 based on the severity of influenza prevalence in that week (measured using the percentage of outpatient visits that show influenza-like symptoms) [12]. These activity levels are also grouped by coarser labels such as minimal (1-3), low (4-5), moderate (6-7), or high (8-10) [13]. We also incorporated the geographic spread index as published by CDC in [14], which categorizes the states based on the internal spatial spread of influenza. We used a number of features associated with each state which are defined by the CDC and can be categorized as follows:

1. Geographical or spatial which included features such as Great Lakes, southeast, mid-Atlantic;

2. Temporal which included features such as activity level (eg, high, moderate, and low) in the *t*th week before the current (at that time) week*,* geographical spread (eg, widespread or local) in the *t*th week prior, whether the number of infections has crossed a threshold, whether the peak has been reached, and similarity with past season. In the description below, these features are denoted by *was1_high* (states with high influenza-like illness activity 1 week prior), *was2_moderate* (states with moderate influenza-like illness activity 2 weeks prior), *was52_high* (states with high activity 52 weeks prior), and so on. These features capture the spatial, temporal, and severity aspects of the reported cases. A full list of attributes and their description is presented in Multimedia Appendix 1.

We used data corresponding to weeks from 2014 to 2017. To generate narratives for a particular week, we use data from these reports for that week, the previous 3 weeks, and the data from 52 weeks prior to generate the temporal data for each state. This was expressed as a data matrix *D* containing the characteristics number of regions as rows (*n*=51 representing 50 states and the District of Columbia) and number of features as columns (*m*=42 spatial, temporal, or severity features). Therefore, the data matrix for a given week had *m*×*n*=2142 entries.

### Problem Formulation

Let *D_n×m_* be the data matrix, where each row corresponds to a state and each column to a feature, and *D_ij_*=1 if state *i* has feature *j*. Let *U*={*e*_1_*,..., e_n_*} be the universe of elements, in our case, the set of all states. Let *D_j_*={*i*: *D_ij_*=1} denote the set of elements having feature *j*. Let *S*(*j*_1_,..., *j_k_*)= 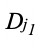 ∩...∩ 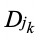 denote the set of elements that have features (*j*_1_,..., *j_k_*) (denoted by ***j***), referred to as a conjunctive clause. The clause S(***j***) has length *k*, meaning that it is formed by the intersection of *k* features.

Given a target set *T* ⊆ *U*, we consider expressions of *T* in terms of unions and differences, ie,


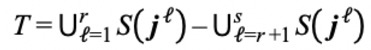
, (**1**)

with an associated cost


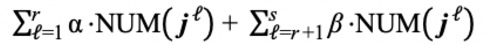
, (**2**)

where and *α* and *β* are the constant parameters associated with positive,


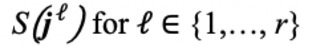
, (**3**)

and negative clauses,


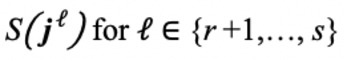
, (**4**)

respectively, and


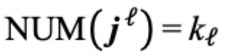
, (**5**)

denotes the number of features involved in a clause


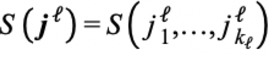
. (**6**)

The negative clauses describe the elements which need to be removed from the set of positive clauses, in order to exactly cover the elements of *T*.

Given a subset *T* ⊆ *U* (referred to as a target set), and a data set *D*, the minimum description length problem involves finding a set of tuples ***j*^1^**,..., ***j^s^***, such that *T* is represented in terms of unions and differences and the associated cost (represented by equation 2) is minimized.

In order to make the descriptions interpretable, we will restrict the sizes of these clauses (ie, the number 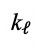 of columns whose intersection is allowed); herein, we will focus on 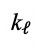
≤2, though our approach extends to any *k*.

Our main idea for finding patterns of interest was to explore the space of all target sets and identify those which have low cost descriptions. This was motivated by the minimum description length principle, that forms the basis of many machine learning methods to find such descriptions; we refer to [15,16] for details on this topic.

In some cases, the target set *T* does not have a small description, but we can find a set *T’* which is close to *T* and has a smaller description than *T*. We model this as finding a representation for a subset *T’* such that *T’≈T*, which is formalized as the minimum approximate description length problem. Given a target set *T* ⊆ *U*, a data set *D*, and constant parameters *α*, *β*, *γ*, the minimum approximate description length problem involves finding a set of tuples ***j*^1^**,..., ***j^s^***, for representation of *T’* as unions and differences, such that the symmetric difference of *T* and *T’* is of size at most *γ*|*T*|, and the associated cost is minimized. Since minimum approximate description length is a generalization of minimum description length, we only consider the minimum approximate description length problem in the rest of the paper. The minimum description length and minimum approximate description length problems are both NP-complete, even when 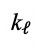
=1, which corresponds to the set cover problem (refer to [17] for discussion on this topic).

### Approach and Implementation

We used an integer programming approach described in Multimedia Appendix 1, which is able to scale well for the problems of interest in epidemic analysis. We used Gurobi optimization software [18] to solve the resulting integer program. The size of the instances encountered results in programs that can be solved very efficiently.

### Generate Set Descriptions.

We considered the set of states with a high activity level in a given week, as a target set *T* and prepared the data matrix *D*. These states had value 1 in the column named high in the matrix. Then, we used our method to compute the succinct descriptions for the target set *T* for the parameters (*α*, *β*, *γ*)=(2, 2, 0). From the minimum description length principle, a set *T* was likely to be an interesting pattern if it had a high compression ratio.

We also studied the impact of the parameter *γ* on the description length. Recall that the parameter controls how accurately we attempt to describe the target set. A larger *γ* would mean greater error but should lead to a more succinct description. The target set *T* was the set of states with high activity in a given week. We ran our method for the given week with target set *T* and, for each value of *γ* ∈ (0.1, 0.2, 0.3).

### Ranking Set Descriptions

It was not known a priori which target sets would give interesting patterns. We searched from a large space of possible target sets corresponding to all clauses with up to *k* terms (ie, sets formed by intersections of up to *k* columns), computed their minimum description length scores, and ranked them based on their compression ratio, and other characteristics.

### Baselines and Evaluation Measures

The work of Xiang et al [19] is directly related to our approach and can be considered as a special case of minimum description length, where only positive clauses are allowed. We referred to this as description by solution. We used the number of clauses used by description by solution and minimum approximate description length for comparison.

We used the compression ratio as a metric for evaluating the performance of our method. The number of clauses used for minimum approximate description length for a target set *T* was *s*. The compression ratio provided by minimum approximate description length was defined as the ratio of the target set size |*T*| to the number of clauses used in the solution to minimum approximate description length, compression ratio=|*T*|/*s*.

We also provided a scoring system to determine the interestingness of a target set. Sets consisting of states with high activity level were likely to be more interesting than those with moderate, low or minimal activity levels; therefore, these were assigned scores of 4, 3, 2, and 1 for high, moderate, low, and minimal activity level, respectively. Next, states exhibiting a sudden change in activity level (eg, from low to high, or vice versa) were considered more interesting than those having no change in activity levels; therefore, we assigned a score of 5 for the former type and 2 for the latter. Then, a set of states with high activity that week and minimal activity 1 week prior had a score of 9, while a set of states with minimal activity that week and minimal activity 1 week prior had a score of 3. This process is described in detail in Multimedia Appendix 1. The score assigned to each target set or description measured its interestingness.

## Results

### Generate Set Descriptions

The text descriptions (manually generated), in Table 1 correspond to solutions computed using our method. The mean compression ratio was 2.63. This showed that our method could easily find succinct descriptions for different kinds of target sets.

Qualitatively, some descriptions (Table 1) involved large target sets (eg, February 18, 2017 and January 3, 2015 which correspond to 27 and 29 states, respectively). The CDC descriptions for these weeks were long lists, which were unlikely to give useful insights or identify any patterns. The description for the week of January 3, 2015 was succinct. Almost all the states with high or moderate activity level in the previous week had high activity in that week, 3 new states that were not experiencing high or moderate activity in the previous week had high activity, and Florida and Georgia experienced a sharp decline in activity levels within the week.

**Figure 1:**
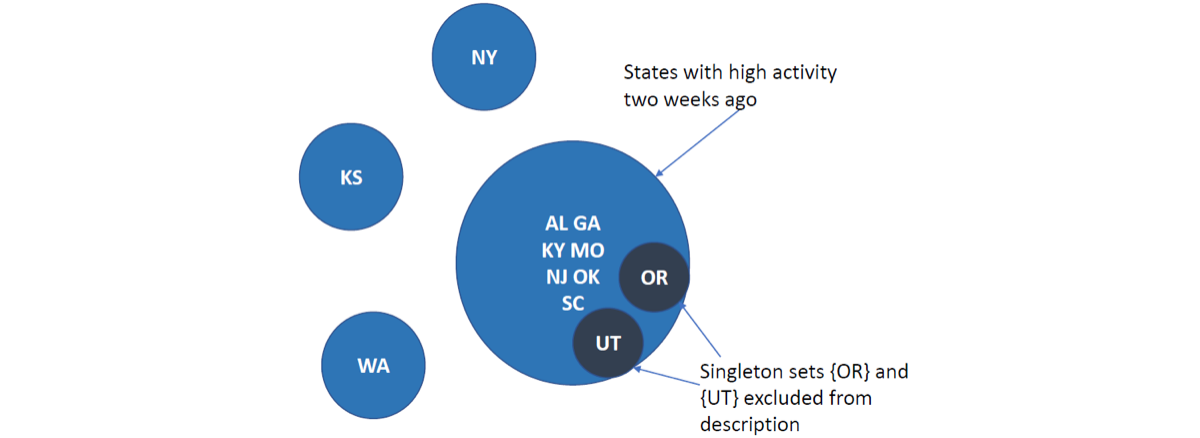
The set representation of the description for week of January 21, 2017. Each circle is a set and the states in the set are listed with their respective abbreviations. The states in the blue region correspond to the target set T. Oregon and Utah are the singleton subsets (in dark blue) with high influenza-like illness activity two weeks prior but not in that week. AL: Alabama; GA: Georgia; ILI: influenza-like illness; KY: Kentucky; KS; Kansas; MO: Missouri; NJ: New Jersey; NY: New York; OK: Oklahoma; OR: Oregon; SC: South Carolina; UT: Utah; WA: Washington.

We also noted that some of the descriptions may not be insightful. For instance, the description for the week of April 8, 2017 was simply a list of 2 states; it is possible that there were no common characteristics between the 2 states, so this was the most succinct. The description for the week of February 18, 2017 was quite complicated. It combined 3 sets of states with different activity levels in different times in the past. Figure 1 shows that a set of 10 states with high influenza-like illness for the week of January 21, 2017 was represented using 6 clauses. The compression ratio achieved was 1.67 as we only use 6 clauses instead of listing 10 state names. However, automated generation of such descriptions will allow a human expert to filter and select appropriate descriptions, instead of creating them from scratch.

**Figure 2:**
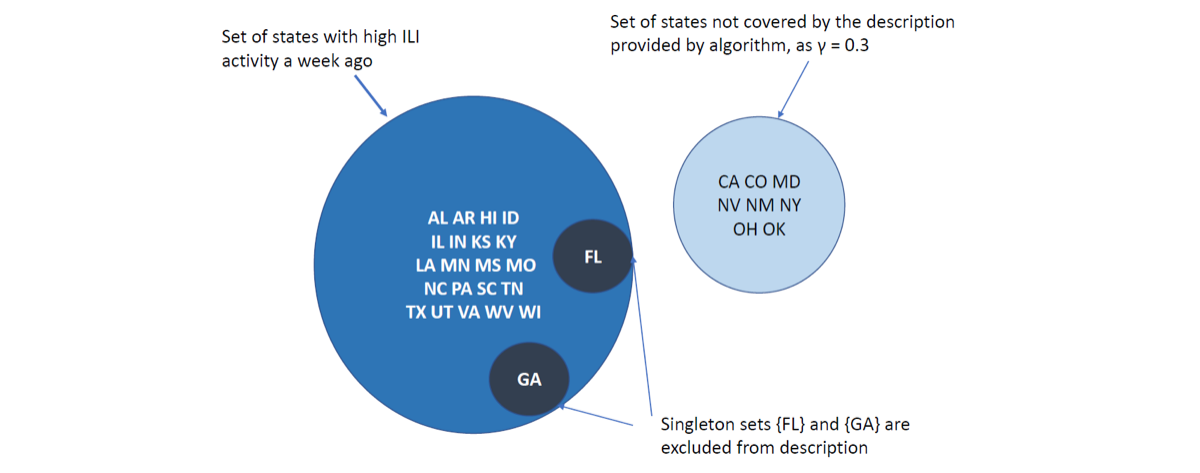
The set representation of description of set of states with high influenza-like illness activity on January 3, 2015. The blue set corresponds to the states with high activity 1 week prior. The dark blue colored singletons Florida and Georgia are subsets of the blue set but do not have high activity in the current week. The light blue colored set consists of the states omitted from the description due to relaxation. AL: Alabama; AR: Arkansas; CA: California; CO: Colorado; HI: Hawaii; ID: Idaho; IL: Illinois; IN: Indiana; KS: Kansas; KY: Kentucky; LA: Louisiana; MD: Maryland; MN: Minnesota; MS: Mississippi; MO: Missouri; NV: Nevada; NM: New Mexico; NY: New York; NC: North Carolina; OH: Ohio; OK: Oklahoma; PA: Pennsylvania; SC: South Carolina; TN: Tennessee; TX: Texas; UT: Utah; VA: Virginia; WV: West Virginia; WI: Wisconsin.

The compression ratio increased as we increased the relaxation factor (Table 2) γ. Figure 2 shows that a set of 29 states with high influenza-like illness for week January 3, 2015 can be represented using only 3 sets per clauses; although 8 out of the 29 states are omitted from the description (shown in the light blue region), as the relaxation parameter is set to 0.3.

**Table 1:** Description for the set of states with high activity levels.

Week	Descriptions of states with high influenza-like illness activity in the week	Number of clauses	Target set	|*T*|	Compression ratio
January 21, 2017	Kansas, New York, Washington, and states with high activity 2 weeks back excluding Oregon and Utah	6	Alabama, Georgia, Kansas, Kentucky, Missouri, New Jersey, New York, Oklahoma, South Carolina, Washington	10	1.67
February 18, 2017	Alaska, Illinois, Maryland, Minnesota, states with high activity a week prior, states with low activity 2 weeks prior, and states with minimal activity 3 weeks prior excluding Wyoming	7	Alabama, Alaska, Arkansas, Connecticut, Georgia, Illinois, Indiana, Kansas, Kentucky, Louisiana, Maryland, Michigan, Minnesota, Mississippi, Missouri, New Jersey, New Mexico, New York, North Carolina, Oklahoma, Pennsylvania, Rhode Island, South Carolina, South Dakota, Tennessee, Texas, Virginia	27	3.86
March 25, 2017	States with high activity for last 2 weeks, excluding Louisiana, Mississippi and Texas	4	Alabama, Arkansas, Georgia, Kansas, Kentucky, North Carolina, Oklahoma, South Carolina, Tennessee, Virginia	10	2.50
April 8, 2017	Kentucky, South Carolina	2	Kentucky, South Carolina	2	1.00
January 3, 2015	California, Nevada, New York, and states with high or moderate activity levels a week prior excluding Florida and Georgia	7	Alabama, Arkansas, California, Colorado, Hawaii, Idaho, Illinois, Indiana, Kansas, Kentucky, Louisiana, Maryland, Minnesota, Mississippi, Missouri, Nevada, New Mexico, New York, North Carolina, Ohio, Oklahoma, Pennsylvania, South Carolina, Tennessee, Texas, Utah, Virginia, West Virginia, Wisconsin	29	4.14

**Table 2:** Impact of varying relaxation factor γ on the description and compression ratio using 2 examples.

Week, *γ*		Description	Clauses, number	Compression ratio
**January 21, 2017**				
	0	Kansas, New York, Washington, and states with high activity 2 weeks prior, excluding Oregon and Utah	6	1.67
	0.1	Kansas, Washington, and states with high activity 2 weeks prior, excluding Oregon and Utah	5	2
	0.2	New York and states with high activity 2 weeks back, excluding Oregon and Utah	4	2.5
	0.3	States with high activity 2 weeks back, excluding Oregon and Utah	3	3.33
**January 3, 2015**				
	0	California, Nevada, New York, and states with high or moderate activity levels a week prior, excluding Florida and Georgia	7	4.14
	0.1	New York, and states with high or moderate activity levels a week prior, excluding Florida and Georgia	5	5.8
	0.2	States with high or moderate activity levels a week prior, excluding Florida and Georgia	4	7.25
	0.3	States with high activity level a week prior, excluding Florida and Georgia	3	9.67

### Ranking Set Descriptions

We found that the top scoring narratives were generally trends. An example of trend found by our method was a gradual increase in activity levels over consecutive weeks; the states Alabama, Georgia, Mississippi, and Tennessee had high activity in the week of March 12, 2016, had moderate activity the previous week, and had minimal activity 2 weeks prior. Another trend was stable high activity for consecutive weeks; in the week ending January 27, 2018, New Jersey, New Mexico, Virginia, Washington, and Wyoming, and states with high activity 4 weeks earlier, excluding Nebraska and Tennessee, had high activity levels for 3 consecutive weeks. Another trend was a gradual decrease in influenza-like illness activity over consecutive weeks; for the week of February 1, 2014, the activity levels in North Carolina decreased from high to moderate to low in 3 consecutive weeks.

Examples of surprise events identified by our methods were (1) the activity level in North Carolina, New Mexico, South Dakota, and Wyoming jumped from low to high within 1 week, for the week ending February 4, 2017 and (2) the activity level in New Hampshire and Tennessee changed from high to low within 1 week, for the week ending February 2, 2013.

**Table 3:** Interestingness scores.

Week	*α*, *β*, *γ*	Target set or pattern	Description	Score
January 27, 2018	(0, 2, 2)	States with high activity the specified week, low activity 2 weeks prior, and moderate activity 3 weeks prior	Hawaii, Maryland, North Carolina, Ohio	14
		States with moderate activity 1 week prior, minimal activity 2 weeks prior, and low activity 3 weeks prior	North Dakota	13
		States with low activity 2 weeks prior, moderate activity 3 weeks prior, and minimal activity 4 weeks prior	Maryland, North Carolina, Ohio	7
February 25, 2017	(0.3, 2, 4)	States with high activity 1 week prior, low activity 2 weeks prior, and moderate activity 3 weeks prior	Iowa	14
		States that had moderate activity levels 1 week prior, minimal activity levels 3 weeks prior, and minimal activity levels 4 weeks prior	Massachusetts, Ohio, Wisconsin	8

### Comparison With Baselines

**Figure 3:**
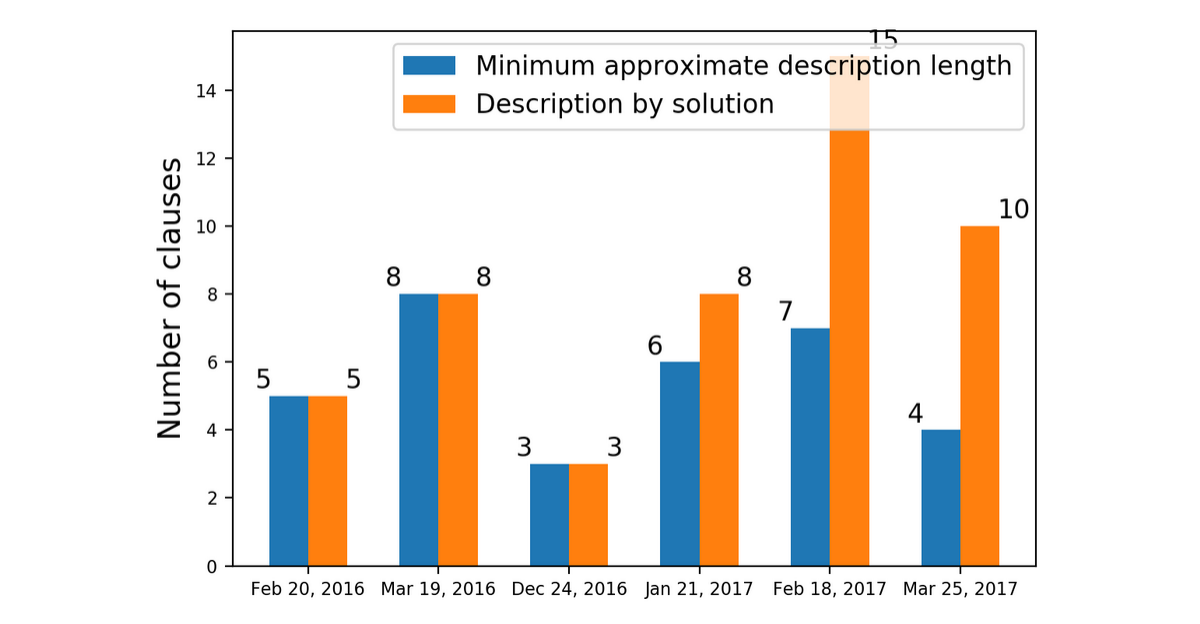
Solution comparison: minimum approximate description length versus description by solution.

Minimum approximate description length provided summaries at less cost than those provided by description by solution for the weeks of January 21, 2017; February 18, 2017; and March 3, 2017 (Figure 3). For the remaining weeks, minimum approximate description length provided summaries at a cost equivalent to those provided by description by solution.

## Discussion

### Principal Findings and Previous Work

There has been a lot of previous work [19-22] on finding spatio-temporal patterns in different data sets. These have typically used unsupervised machine learning methods, and we refer the readers to [20,21] for surveys on different algorithms and their applications to various data sets. As is the case with other unsupervised methods, the specific technique depends on the application. We note that mining patterns from transactional data has been successfully used in many areas, such as analysis of retail transaction data [23], biomedical data analysis [19,24] and information retrieval [25]. The approach of finding patterns based on compression and small description have been found to be useful in many settings [22,26-28]. We found that our description length-based approach gives useful insights into spatio-temporal patterns in incidence of influenza-like illness, especially when negative clauses are allowed. However, no prior methods handle negative clauses, to the best of our knowledge. In addition to negative clauses, we also found that the relaxed versions can also significantly reduce the complexity of descriptions in many cases.

Our ranking method also provides a systematic approach to identify trends and surprises in the spread of influenza-like illness. However, the descriptions of high score are not always intuitive or interesting, which is often the case with unsupervised machine learning methods. Instead, our ranking-based approach (or other variations of it) could help provide new insights to a domain expert, who might be able to find interesting spatio-temporal patterns more easily. Thus, such an approach could be a first step in processing epidemic incidence data. We believe that including more characteristics for the data (ie, more columns in the data matrix *D*) can help find more succinct descriptions. Furthermore, the integer programming–based approach is quite powerful, and more constraints can be easily added to generate descriptions with specific kinds of properties. Though the descriptions reported here were generated manually based on the outputs, the outputs are well structured and could conceivably be generated using natural language processing techniques easily.

Comparing the performance of our method with 2 other pattern detection methods in the literature, though, as mentioned earlier, which do not consider negative clauses, the first method, called Apriori [23] is a very popular approach for association rule mining and pattern detection in a database containing transactions. Each transaction is seen as a set of items called itemset. The Apriori algorithm finds the frequent item sets in the database, the item sets that appear frequently among the transactions of the database. We observed that the rules generated by Apriori using Weka [29] are trivial in nature and are not highly informative.

The work of Xiang et al [19] (description by solution) can be considered as a special case of minimum description length, where only positive clauses are allowed. Xiang et al [19] give a logarithmic approximation for the description by solution problem for such instances. We implement an integer linear program to solve this problem exactly. By comparing the solutions provided by minimum approximate description length with that of description by solution, we demonstrated the benefit of allowing differences in generating compact descriptions. We note that using additional attributes for the regions might allow for more succinct descriptions.

Our methodology could be easily extended to other diseases and applications involving spatio-temporal data, since the method can handle very general kinds of features and clauses formed by them. The ranking method would have to be designed based on the specific domain. Also, we expect our method could scale to much larger data sets easily.

### Limitations

The feature values are real numbers (eg, the similarity with a past season can be a correlation metric) not binary. One way to handle this issue would be to map the nonbinary values to binary using discretization of the weights. Since we limited our focus to only meaningful features, our current approach explores target sets with temporal properties over small time intervals. In the case of an increase in number of features by a few orders of magnitude than we considered, the integer linear program may not be able to scale well. One way to address this problem would be to design scalable heuristics that give some theoretical or experimental guarantees.

### Conclusion

Automated generation of interesting spatio-temporal patterns and trends is an important problem, and can be especially useful to public health experts, as well as the general public. Our approach, based on techniques from pattern mining, provide a short-list of patterns in influenza-like illness data from the CDC. We found that sets with high compression ratio tend have common characteristics, which are often interesting. This is, however, an unsupervised machine learning method, and needs to be verified manually. Our ranking method is one way to select interesting patterns in an automated manner. The techniques developed in this paper could potentially be applied for other diseases, and other public health domains.

## Appendix

Multimedia Appendix 1 Additional material.

## Abbreviations

CDC: Centers for Disease Control and Prevention
