# Supplementary material for: An Automated Approach for Finding Spatio-Temporal Patterns of Seasonal Influenza in the United States: Algorithm Validation Study
Source: JMIR Public Health Surveill. 2020 Sep 4;6(3):e12842. doi: 10.2196/12842 (PMC7501584; doi:10.2196/12842)
Supplement: Multimedia Appendix 1 [file publichealth_v6i3e12842_app1.docx]

# Appendix

## Example to understand the notation

An example of the input data is shown in Table 1, in the form of a matrix *D*. It has six rows (corresponding to regions), denoted here by {*e*_1_*, e*_2_*, e*_3_*, e*_4_*, e*_5_*, e*_6_}. *D* has twelve columns, denoted here by {*f*_1_*, f*_2_*, f*_3_*, f*_4_*, f*_5_*, f*_6_}, in addition to one column for each row. The first six columns correspond to features, e.g., “South East” (which means the region is in South Eastern US) and “Was1_high” (which means the region was high last week).

|  | (South East) $f_{1}$ | (Mid- Atlantic) $f_{2}$ | (Was1- high) $f_{3}$ | (Was1- low) $f_{4}$ | (Was2- low) $f_{5}$ | (Was52-high) $f_{6}$ | (e1) $f_{7}$ | (e2) $f_{8}$ | (e3) $f_{9}$ | (e4) $f_{10}$ | (e5) $f_{11}$ | (e6) $f_{12}$ |
| --- | --- | --- | --- | --- | --- | --- | --- | --- | --- | --- | --- | --- |
| $e_{1}$ | 1 | 1 | 1 | 0 | 0 | 1 | 1 | 0 | 0 | 0 | 0 | 0 |
| $e_{2}$ | 0 | 0 | 0 | 1 | 0 | 1 | 0 | 1 | 0 | 0 | 0 | 0 |
| $e_{3}$ | 0 | 0 | 0 | 0 | 1 | 0 | 0 | 0 | 1 | 0 | 0 | 0 |
| $e_{4}$ | 1 | 1 | 1 | 0 | 1 | 0 | 0 | 0 | 0 | 1 | 0 | 0 |
| $e_{5}$ | 1 | 1 | 0 | 0 | 1 | 1 | 0 | 0 | 0 | 0 | 1 | 0 |
| $e_{6}$ | 0 | 0 | 1 | 0 | 0 | 1 | 0 | 0 | 0 | 0 | 0 | 1 |

Table 1: Sample input, represented as a matrix D of dimensions 6 × 12. The rows (corresponding to states) are elements of U, and the first six columns are features. The next six columns are added for computational reasons.

Let *U* = {*e*_1_*, e*_2_*, e*_3_*, e*_4_*, e*_5_*, e*_6_}. In the matrix D in Table 1, *D*_2_ = {*e*_1_*, e*_4_*, e*_5_} and

*D*_5_ = {*e*_3_*, e*_4_*, e*_5_} are examples of sets having features *f*_2_ and *f*_5_, respectively. Then, *S*(2*,* 5) is a clause, which represents the set *S*(2*,* 5) = *D*_2_ ∩ *D*_5_ = {*e*_4_*, e*_5_}. Note that the column *D*_7_ = {*e*_1_} is also a clause, represented by *S*(7).

We continue our example from Table 1. An example of a target set $T$ is the set of regions that have feature $f_{1}$, i.e., $T=\{e_{1},e_{4},e_{5}\}$. $T$ can be expressed as combinations of different kinds of clauses. For instance, $T=S\left( 7 \right)\cup S\left( 10 \right)\cup S\left( 11 \right)$. This representation has cost$3\alpha$. Note that $D_{2}=D_{1}=T$, so $T$ can also be expressed simply as $T=S\left( 2 \right)$; this has cost $\alpha$. Finally, we can represent $T$ the target set as unions and differences of clause as $T=S\left( 2,5 \right)\cup S\left( 3,6 \right)-S\left( 3,4,6 \right)$, since $S\left( 2,5 \right)=D_{2}\cap D_{5}=\{e_{4},e_{5}\}$, $S\left( 3,6 \right)=D_{3}\cap D_{6}=\{e_{1},e_{6}\}$, and $S\left( 3,4,6 \right)=D_{3}\cap D_{4}\cap D_{6}=\{e_{6}\}$.

However, this representation has cost $2\alpha+ 2\alpha+ 3\beta=4\alpha+3\beta$.

Continuing our example, let $T=\{e_{1},e_{3},e_{4}\}$. Let us restrict ourselves on to the use of features $f_{1},\ldots,f_{6}$ in the clauses to represent $T$ in required form. Then, $S\left( 1 \right)\cup S\left( 5 \right) = \{e_{1},e_{3},e_{4},e_{5}\}$ (alternatively, $S\left( 2 \right)\cup S\left( 5 \right)$) covers all elements of $T$ with just one extra element $e_{5}$. Then, to exactly represent $T$, there isn’t any negative clause to pick.

## Integer Program (IP)

The $MinDesc$ problem requires exploring over the space of all possible representations for a set $T$, and choosing one that has the minimum cost. The $MinApproxDesc$ problem has the additional requirement of ensuring that a large part of $T$ is represented. These are both computationally very hard. Formally, these problems are NP-complete, even when $k_{\mathcal{l}}=1$ (i.e., each clause only has one feature. We refer to [18] for an introduction to this topic. Here, we solve these problems using integer programming, which is a powerful and general technique for solving combinatorial optimization problems. We describe our formulation as an integer program (IP), and how it is solved.

## Problem Formulation (Full version)

Let *D_n_*_×_*_m_* be the data matrix, where each row corresponds to a state and each column to a feature, and *D_ij_* = 1 if state *i* has that feature. Let *U* = {*e*_1_*, ..., e_n_*} be the universe of elements, in our case, the set of all states. Let *D_j_* = {*i* : *D_ij_* = 1} denote the set of elements having feature *j*. Let *S*(*j*_1_*, . . . , j_k_*) = *D_j1_  ∩ …∩ D_jk_* denote the set of elements that have features *j*_1_*, …, j_k_*; referred as a conjunctive *clause*. The clause *S*(*j*_1_*, . . . , j_k_*) has length *k*, meaning that it is formed by the intersection of *k* features; note that this is not the same as the number of elements in S. We associate a cost function *c*(*j*_1_*, . . . , j_k_*); the simplest would be *c*(*j*_1_*, . . . , j_k_*) = *αk* for a constant *α*.

Table 1: Definitions and notations

| **Term** | **Definition** | **Description** |
| --- | --- | --- |
| $U$ | $\{e_{1},\ldots,e_{n}\}$ | Universe set |
| $T$ | $T \subseteq U$ | Target set |
| $D_{j}$ | $\{i:D_{ij}=1\}$ | Set of elements having feature $j$. |
| $\boldsymbol{j}^{\mathcal{l}}$ | $j_{1},\ldots,j_{k}$ | List of features $j_{1},\ldots,j_{k}$ in $\mathcal{l}^{th}$ clause. |
| $S\left( \boldsymbol{j}^{\mathcal{l}} \right)$ | $D_{j_{1}}\cap\ldots\cap D_{j_{k}}$ | Set of elements that have all features in list $\boldsymbol{j}^{\mathcal{l}}$ |

Given a target set *T* ⊆ *U*, we consider expressions of *T* in terms of unions and differences of such clauses, having the following form

$$T = \bigcup_{\mathcal{l}=1}^{r} S\left( j_{1}^{\mathcal{l}},\ldots,j_{k_{\mathcal{l}}}^{\mathcal{l}} \right) - \bigcup_{\mathcal{l}=r+1}^{s} S\left( j_{1}^{\mathcal{l}},\ldots,j_{k_{\mathcal{l}}}^{\mathcal{l}} \right),$$

with an associated cost of

$$\sum_{\mathcal{l}=1}^{r} \alpha k_{\mathcal{l}}+\sum_{\mathcal{l}=r+1}^{s} \beta k_{\mathcal{l}},$$

where and $\alpha$ and $\beta$ are the constant parameters associated with positive and negative clauses. The clauses $S\left( j_{1}^{\mathcal{l}},\ldots,j_{k_{\mathcal{l}}}^{\mathcal{l}} \right)$ corresponding to$\mathcal{l}=1,\ldots,r$ are “positive” clauses, and $\alpha$ is the cost for each such clause. The clauses corresponding to $\mathcal{l}=r+1,\ldots,s$ are “negative” clauses and describe elements which need to be removed from the set of positive clauses, in order to exactly cover the elements of $T$; with the cost parameter $\beta$. For succinctness, we use $\boldsymbol{j}^{\mathcal{l}}=\left( j_{1}^{\mathcal{l}},\ldots,j_{k_{\mathcal{l}}}^{\mathcal{l}} \right)$ to denote such a tuple, and $S\left( \boldsymbol{j}^{\mathcal{l}} \right)=S\left( j_{1}^{\mathcal{l}},\ldots,j_{k_{\mathcal{l}}}^{\mathcal{l}} \right)$ as the corresponding clause. We use $NUM\left( \boldsymbol{j}^{\mathcal{l}} \right)=k_{\mathcal{l}}$ to denote the number of features involved in such a clause. Then, the representation for $T$

can be written as

$$T=\bigcup_{\mathcal{l}=1}^{r} S\left( \mathbf{j}^{\mathcal{l}} \right)-\bigcup_{\mathcal{l}=r+1}^{s} S\left( \mathbf{j}^{\mathcal{l}} \right),$$

with an associated cost of

$$\sum_{\mathcal{l}=1}^{r} \alpha\cdot NUM\left( \boldsymbol{j}^{\mathcal{l}} \right)+\sum_{\mathcal{l}=r+1}^{s} \beta\cdot NUM\left( \boldsymbol{j}^{\mathcal{l}} \right).$$

Finally, we use $\mathcal{C}^{\mathcal{k}}=\{\boldsymbol{j}=\left( j_{1},\ldots,j_{k^{'}} \right):k^{'}\leq k\}$ to denote the set of all such tuples of length at most $k$; for an element $i$, let $\mathcal{C}_{\mathcal{i}}^{\mathcal{k}}=\{\boldsymbol{j}=\left( j_{1},\ldots,j_{k^{'}} \right):k^{'}\leq k,i\in S\left( \boldsymbol{j} \right)\}$ denote the set of tuples such that the corresponding clauses contain $i$.

Given a subset $T \subseteq U$ (referred to as a “target” set), and a dataset $D$, the $MinDesc\left( T,D \right)$ problem involves finding a set of tuples $\boldsymbol{j}^{\boldsymbol{1}},\ldots,\boldsymbol{j}^{\boldsymbol{s}}$, such that

$$T=\bigcup_{\mathcal{l}=1}^{r} S\left( \boldsymbol{j}^{\mathcal{l}} \right)- \bigcup_{\mathcal{l=}r+1}^{s} S\left( \boldsymbol{j}^{\mathcal{l}} \right),$$

and the associated cost $\sum_{\mathcal{l}=1}^{r} \alpha\cdot NUM\left( \boldsymbol{j}^{\mathcal{l}} \right)+\sum_{\mathcal{l=}r+1}^{s} \beta\cdot NUM\left( \boldsymbol{j}^{\mathcal{l}} \right)$ is minimized. In order to make the descriptions interpretable, we will restrict the sizes of these clauses, i.e., the number $k_{\mathcal{l}}$ of columns whose intersection is allowed; here, we will focus on $k_{\mathcal{l}}\leq2$, though our approach extends to any $k$.

Our main idea for finding patterns of interest is to explore the space of all target sets and identify those which have low cost descriptions. This is motivated by the *Minimum Description Length* (MDL) Principle, that forms the basis of many machine learning methods to find such descriptions; we refer to [15, 30] for details on this topic.

Specifically, we find a *succinct* representation of the set $T$ of elements, in terms of combinations of different features. For instance, suppose the set of states which are currently experiencing high activity are precisely those in the East and South.

These could be described in two alternative ways:

(a) by just listing all the states (e.g., VA, NC, MD, etc.) or,

(b) as just Eastern and Southern states.

The latter is preferred because of its succinctness.

Note that for each element in $U$ there is a feature in our data matrix. This is required to be able to represent any given target set $T$ in terms of unions and differences of clauses.

In some cases, the target set $T$ does not have a small description, but we can find a set $T^{‘}$ which is *close* to $T$, and has a smaller description than $T$. We model this as finding a representation for a subset $T^{’}$ such that $T^{’}\approx T$, which is formalized as the $MinApproxDesc$ problem: Given a target set $T\subseteq U$, a dataset $D$, and constant parameters $\alpha, \beta, \gamma$, the $MinApproxDesc\left( T,D \right)$ problem involves finding a set of tuples $\boldsymbol{j}^{\boldsymbol{1}},\ldots,\boldsymbol{j}^{\boldsymbol{s}}$, such that

$$T^{'}=\bigcup_{\mathcal{l}=1}^{r} S\left( \boldsymbol{j}^{\mathcal{l}} \right)-\bigcup_{\mathcal{l}=r+1}^{s} S\left( \boldsymbol{j}^{\mathcal{l}} \right),$$

$\left| \{i:i\in T\setminus T^{'}\cup T^{'}\setminus T\} \right|\leq\gamma\left| T \right|$, and the associated cost $\sum_{\mathcal{l}=1}^{r} \alpha\cdot NUM\left( \boldsymbol{j}^{\mathcal{l}} \right)+\sum_{\mathcal{l}=r+1}^{s} \beta\cdot NUM\left( \boldsymbol{j}^{\mathcal{l}} \right)$ is minimized. We refer to $\alpha, \beta, \gamma$ as the parameters associated with the relaxed version.

In other words, the $MinApproxDesc$ problem finds a representation for a subset $T^{'}$ that is “close” to $T$. Since *MinApproxDesc* is a generalization of *MinDesc*, we only consider the *MinApproxDesc* problem in the rest of the paper.

The$MinDesc$ and $MinApproxDesc$ problems are both NP-complete, even when $k_{\mathcal{l}}=1$, which corresponds to the *set cover* problem (we refer to [17] for discussion on this topic). Here, we use an integer programming approach described in the Appendix, which is able to scale well for the problems of interest in epidemic analysis. We use the Gurobi optimization software [18] to solve the resulting Integer program. The size of the instances encountered results in programs that can be solved very efficiently. So, we expect our method will scale to much larger datasets easily.

### Measures

We use the compression ratio as a metric for evaluating the performance of our method. Let the number of clauses used in description by *MinApproxDesc* for a target set *T* be *s*. The compression ratio provided by *MinApproxDesc* is defined as the ratio of the target set size |*T*| to the number of clauses used in the description by solution to *MinApproxDesc*,

$$compression ratio=\frac{|T|}{s}$$

### Descriptions for sets of high activity levels.

We consider the set of states with a high activity level in the current week, as a target set *T*. We prepare the data matrix *D* for the current week. These states have value 1 in the column ‘high’ of the matrix. Then, we use our method to compute the succinct descriptions for the target set *T* for the parameters $(\alpha, \beta, \gamma)$ = (2, 2, 0). From the MDL principle, a set $T$ is likely to be an interesting pattern if it has a high compression ratio.

We also study the impact of the parameter $\gamma$ on the description length. Recall that the parameter $\gamma$ controls how accurately we attempt to describe the target set. A larger $\gamma$ would mean greater error, but should lead to a more succinct description. The target set *T* is the set of states with high activity in the current week. We run our method for a given week with target set *T* and, for each value of $\gamma\in\{0.1, 0.2, 0.3\}.$

### Generation of descriptions by ranked order.

It is not known a priori which target sets would give interesting patterns. We search from a large space of possible target sets corresponding to all clauses with up to $k$ terms (i.e., sets formed by intersections of up to k columns), compute their MDL scores, and rank them based on their compression ratio, and other characteristics.

Sets consisting of states with high activity level are likely to be more interesting than those with moderate, low or minimal activity levels; therefore, these are assigned scores 4, 3, 2, 1 respectively (i.e., 4 for sets with high activity level, and so on). Next, states exhibiting a sudden change in activity level (e.g., from low to high, or vice versa) are more interesting than those having no change in activity levels we assign a score of 5 for the former type, and 2 for the latter. Then, “a set of states with high this week and minimal 1 week ago” has a score of 9, while “a set of states with minimal this week and minimal 1 week ago” has a score of 3. This process is described in detail in the Appendix. The score assigned to each target set/ description measures its “interestingness”. We say that a set of states has a “trend” if it exhibits a gradual increase or decrease in activity level. We refer to a sudden rise or drop in activity levels (by at least two levels, say, from high to low, moderate to minimal, etc.) within a week's time as a “surprise”.

### A. Integer Program (IP) for $MinDesc$ Problem

We start by specifying the variables and the objective function. Recall the notion of a tuple of features $\boldsymbol{j}^{\mathcal{l}}=\left( j_{1}^{\mathcal{l}},\ldots,j_{k_{\mathcal{l}}}^{\mathcal{l}} \right)$, and a clause $S\left( \boldsymbol{j}^{\mathcal{l}} \right)=S\left( j_{1}^{\mathcal{l}},\ldots,j_{k_{\mathcal{l}}}^{\mathcal{l}} \right)$. For $\boldsymbol{j}\in\mathcal{C}^{\mathcal{k}}$, let $y\left( \boldsymbol{j} \right)$ be an indicator variable for $S\left( \boldsymbol{j} \right)$ being used as a positive clause, i.e., $y\left( \boldsymbol{j} \right)=1$ in this case, and 0 otherwise. Similarly, $z\left( \boldsymbol{j} \right)$ is the indicator variable for $S\left( \boldsymbol{j} \right)$ being used as a negative clause.

We have the following integer program (IP):


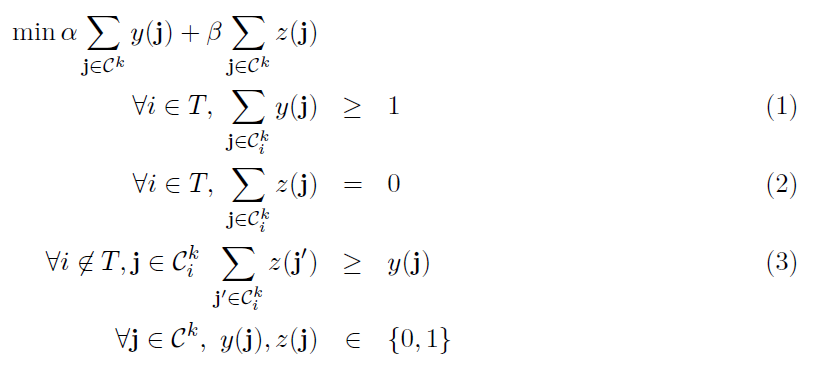


We observe that this is a valid set of inequalities. The constraints (1) indicate that each element $i\in T$ must be part of a positive clause. Since $\mathcal{C}_{\mathcal{i}}^{\mathcal{k}}$ is the set of all clauses of size at most $k$, containing element $i$, this constraint implies that at least one of these clauses must be picked as a positive clause, which means $y\left( \boldsymbol{j} \right)$ must be 1 for some $\boldsymbol{j}\in\mathcal{C}_{\mathcal{i}}^{\mathcal{k}}$. No element $i\in T$ must be part of a negative clause, else it will not be part of the representation. This is captured in the constraints (2), which ensures that $z\left( \boldsymbol{j} \right)=0$ for each $\boldsymbol{j}\in\mathcal{C}_{\mathcal{i}}^{\mathcal{k}}$. Finally, if there exists a clause $\boldsymbol{j}$ containing an element $i\notin T$, with $y\left( \boldsymbol{j} \right)=1$, the solution must contain a negative clause $\boldsymbol{j}^{'}$ to “remove” it. Therefore, we need $z\left( \boldsymbol{j}^{'} \right)=1$ for some $\boldsymbol{j}^{'}\in\mathcal{C}_{\mathcal{i}}^{\mathcal{k}}$. This is captured through constraints (3), which ensures that $z\left( \boldsymbol{j}^{'} \right)=1$ if $y\left( \boldsymbol{j} \right)=1$ for any $\boldsymbol{j}\in\mathcal{C}_{\mathcal{i}}^{\mathcal{k}}$, for some $i\notin T$.

In our work, we focus on $k=2$, since the descriptions become very complex and hard to interpret otherwise. The data matrix $D$ is constructed using the ILI data

for multiple seasons. The space of tuples $\mathcal{C}^{\mathcal{k}}$ is constructed from $D$, and the resulting IP is solved using the Gurobi optimization software [19]. For the scale of problems considered here, this solver runs within seconds, and returns the variables $y\left( \boldsymbol{j} \right),z\left( \boldsymbol{j} \right)$ which are set to 1. These are then used to construct the representations discussed in the Results.

### Appendix B: IP for $MinApproxDesc$ Problem

Recall that the $MinApproxDesc$ gives a succinct representation for a set $T^{'}$, which is “close” to $T$. We formalize this by requiring that $\left| T\oplus T^{'} \right|=\left| T-T^{'} \right|+\left| T^{'}-T \right|\leq\gamma\left| T \right|$. We solve this by a slight modification of the above IP. Specifically, we have a variable $x\left( i \right)$ for each element $i\in U$ with the following semantics:

(1) if $i\in T$ is not represented, i.e., $i\in T-T^{'}$, then $x\left( i \right)=1$, and

(2) if $i \notin T$ is represented, i.e., $i\in T^{'}-T$, then $x\left( i \right)=1$.

If neither of these conditions are satisfied, we have $x\left( i \right)=0$. Then, $\sum_{i} x\left( i \right)=\left| T\oplus T^{'} \right|$ measures the difference between $T$ and $T^{'}$. The following IP solves the $MinApproxDesc$ problem.


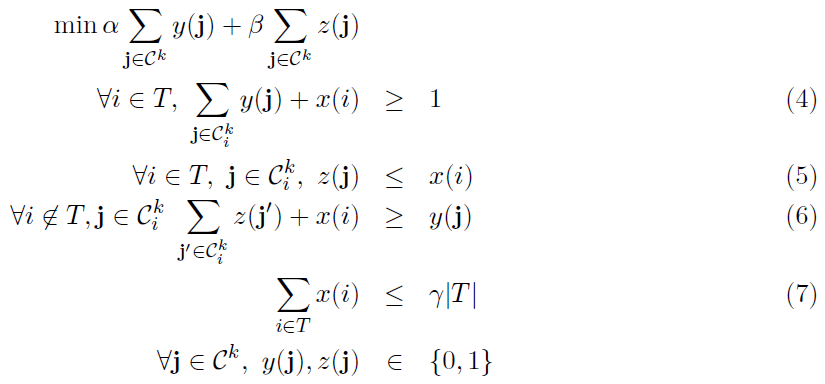


The correctness of the above program follows on the same lines as that for $MinDesc$.

The only difference is that if $x\left( i \right)=1$ for some $i\in T$, that element does not have to be covered. Therefore, unlike inequality (1), $\sum_{\boldsymbol{j}\in\mathcal{C}_{\mathcal{i}}^{\mathcal{k}}} y\left( \boldsymbol{j} \right)\geq1-x\left( i \right)$ is sufficient now. Similarly, $z\left( \boldsymbol{j} \right)$ could now be 1 for such an $i\in T$ if $x\left( i \right)=1$. Therefore, we have inequalities (5) instead of (2). Finally, for $i\notin T$, if it is covered by some $y\left( \boldsymbol{j} \right)=1$, for $\boldsymbol{j}\in\mathcal{C}_{\mathcal{i}}^{\mathcal{k}}$, it does not have to be accounted for, if $x\left( i \right)=1$; therefore, inequalities (6) have $x\left( i \right)$ on the left side, in contrast with (3). We solve the above program in the same way, for different values of $\gamma$, which determines the $y\left( \boldsymbol{j} \right),z\left( \boldsymbol{j} \right)$ variables set to 1. These are then used to construct descriptions for the set $T$.

**Additional Experimental Results**

Table 5: (Full version of Table 2) Description for the set of states with high activity level for certain weeks during 2014–2017. The textual description is written by hand, corresponding to the solutions computed using our method for the values shown. The abbreviations are used for state names [21]. The last column indicates the number of states with a high activity level in that week, for which the description is presented.

| **S.No.** | **Week** | **γ** | **Descriptions of states with high activity level in the week** | **No. of clauses (s) used in description** | **Target Set Size \|T\|** | **Compression ratio** |
| --- | --- | --- | --- | --- | --- | --- |
| 1 | 2016-02-20 | 0 | AZ, MD, NM, TX and UT | 5 | 5 | 1 |
|  |  | 0.1 | AZ, MD, NM, TX and UT | 5 |  | 1 |
|  |  | 0.2 | MD, NM, TX and UT | 4 |  | 1.25 |
|  |  | 0.3 | MD, NM, TX and UT | 4 |  | 1.25 |
| 2 | 2016-03-19 | 0 | AR, HI, NC, NJ, VA, WY and the states with high activity both 1 week and 3 weeks ago | 7 | 8 | 1.14 |
|  |  | 0.1 | AR, HI, NC, NJ, VA, WY and the states with high activity both 1 week and 3 weeks ago | 7 |  | 1.14 |
|  |  | 0.2 | HI, NC, NJ, VA, WY and the states with high activity both 1 week and 3 weeks ago | 6 |  | 1.33 |
|  |  | 0.3 | NC, NJ, VA, WY and the states with high activity both 1 week and 3 weeks ago | 5 |  | 1.6 |
| 3 | 2016-12-24 | 0 | AL, GA and MS | 3 | 3 | 1 |
| 4 | 2017-01-21 | 0 | KS, NY, WA, and states with high activity two weeks back, excluding OR and UT | 6 | 10 | 1.67 |
|  |  | 0.1 | KS, WA, and states with high activity two weeks ago, excluding OR and UT | 5 |  | 2 |
|  |  | 0.2 | NY and states with high activity two weeks back, excluding OR and UT | 4 |  | 2.5 |
|  |  | 0.3 | States with high activity two weeks back excluding OR and UT | 3 |  | 3.33 |
| 5 | 2017-02-18 | 0 | AK, IL, MD, MN, states with high activity a week ago, states with low activity two weeks ago, and states with minimal activity three weeks ago, excluding WY | 7 | 27 | 3.86 |
|  |  | 0.1 | IL, MN, states with high activity a week ago, states with low activity two weeks ago and states with minimal activity three weeks ago, excluding WY | 5 |  | 5.4 |
|  |  | 0.2 | States with high activity a week ago, states with low activity two weeks ago and states with minimal activity three weeks ago, excluding WY | 3 |  | 9 |
|  |  | 0.3 | States with high activity a week ago, excluding WY | 2 |  | 13.5 |
| 6 | 2017-03-25 | 0 | States with high activity for last two weeks, excluding LA, MS and TX | 4 | 10 | 2.5 |
| 7 | 2017-04-08 | 0 | KY and SC | 2 | 2 | 1 |
| 8 | 2014-12-13 | 0 | AR, IL, IN, KS, MN, MO, OK, VA, and states with high activity a week ago | 9 | 13 | 1.44 |
|  |  | 0.1 | AR, IL, IN, KS, MN, MO, VA, and states with high activity a week ago | 8 |  | 1.63 |
|  |  | 0.2 | IL, IN, KS, MN, MO, OK, VA, and states with high activity a week ago | 7 |  | 1.86 |
|  |  | 0.3 | IL, IN, MN, MO, OK, VA, and states with high activity a week ago | 6 |  | 2.17 |
| 9 | 2015-01-03 | 0 | CA, NV, NY, and states with high or moderate activity levels a week ago excluding FL and GA | 7 | 29 | 4.14 |
|  |  | 0.1 | NY, and states with high or moderate activity levels a week ago excluding FL and GA | 5 |  | 5.8 |
|  |  | 0.2 | States with high or moderate activity levels a week ago excluding FL and GA | 4 |  | 7.25 |
|  |  | 0.3 | States with high activity level a week ago excluding FL and GA | 3 |  | 9.67 |
| 10 | 2015-03-14 | 0 | States with high activity both 1 week and 4 weeks ago, excluding CT | 2 | 11 | 5.5 |

Figure 4: Compression ratio of the solution provided by MinApproxDesc. The X-axis corresponds to the week with the S.No. in Table 2. The Y-axis corresponds to the compression ratio of the solution provided by MinDesc. The red horizontal line corresponds to the average compression ratio provided by MinApproxDesc over all the weeks.


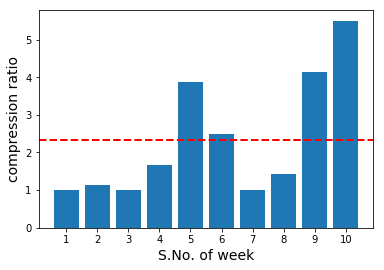


Figure 5 shows an overview of our methodology. In the results section, we present some descriptions generated by our algorithm for a particular target set, followed by a discussion on effects of the parameters. Finally, we present the utility of these methods in automatically identifying certain patterns in the data.

Figure 5: Overview of steps, to generate succinct description of the level of influenza like illnesses (ILI) in different states of USA. The process begins with collecting raw ILI data from CDC website, followed by the creation of a state attribute table — a domain-specific version of the transaction-item matrix D — for a given weekend. We iterate over a space of all potential target sets and solve the $MinApproxDesc$ problem to compute a representation. These are then ranked based on their interestingness score.

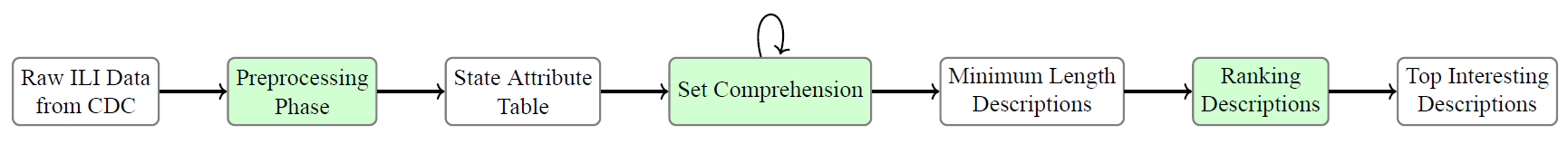


**Dataset Description**

We begin with Influenza-like Illness (ILI) Activity Indicator data available at state level with weekly updates from CDC [22]. In the dataset, each state for each week during a given influenza season, is assigned an activity level from 1 to 10 based on the severity of influenza prevalence in that week (measured using the percentage of outpatient visits that show influenza-like symptoms) [23]. These activity levels are also grouped into coarser labels such as Minimal (1-3), Low (4-5), Moderate (6-7), High (8-10). We use this state level activity data to prepare target sets for a given week (say the states with High level of activity). We also incorporate the geographic spread index as published by CDC in [24], which categorizes the states based on the internal spatial spread of influenza. We use a number of features associated with each state, which are defined by the CDC, and can be categorized as follows:

1. Geographical/ Spatial: Features such as Great Lakes, South East, Mid-Atlantic etc.

2. Temporal: Features such as activity level (e.g., high, moderate and low) in the *t*th week before the current one, geographical spread (e.g., widespread, local) in the *t*th week before the current one, whether the number of infections has crossed a threshold, whether the peak has been reached, and similarity with past season. In the description below, these features will be denoted by “was1_high” (states with high ILI activity 1 week ago), “was2_moderate” (states with moderate ILI activity 2 weeks ago), “was52_high” (states with high activity 52 weeks ago), etc.

These features capture the spatial, temporal, and severity aspects of the reported cases. In general, these values are real numbers, e.g., the similarity with a past season can be a correlation metric, but we assume these have been transformed into binary features (e.g., high/low activity level, which is already available in the data from CDC). The non-binary setting can be mapped to a binary setting, through discretization of the weights. Full list of attributes and their description is presented in the Appendix.

In our experiments, we use data corresponding to weeks for the years 2014 to 2017. To generate narratives for a particular week, we use the data from these reports for the current week, the last three weeks, and the data 52 weeks ago to generate the temporal data for each state. This is expressed as a data matrix D with the following characteristics:

1. Number of regions (states) or rows: 51 (50 states and District of Columbia)
2. Number of features or columns: 42 (spatial, temporal, and severity features)
Therefore, the data matrix $D$ for a week has 2142 entries.

## Data Description

We first present the regions/states used in the dataset along with their abbreviations followed by the list of attributes and their description.

**Description of states (or regions) and Abbreviations**

| **State or Region code** | **State or Region name** | **Abbreviation** | **Region code** |
| --- | --- | --- | --- |
| 91 | New England Region | NENG | 1 |
| 9 | Connecticut | CT | 1 |
| 23 | Maine | ME | 1 |
| 25 | Massachusetts | MA | 1 |
| 33 | New Hampshire | NH | 1 |
| 44 | Rhode Island | RI | 1 |
| 50 | Vermont | VT | 1 |
| 92 | Mideast Region | MEST | 2 |
| 10 | Delaware | DE | 2 |
| 11 | District of Columbia | DC | 2 |
| 24 | Maryland | MD | 2 |
| 34 | New Jersey | NJ | 2 |
| 36 | New York | NY | 2 |
| 42 | Pennsylvania | PA | 2 |
| 93 | Great Lakes Region | GLAK | 3 |
| 17 | Illinois | IL | 3 |
| 18 | Indiana | IN | 3 |
| 26 | Michigan | MI | 3 |
| 39 | Ohio | OH | 3 |
| 55 | Wisconsin | WI | 3 |
| 94 | Plains Region | PLNS | 4 |
| 19 | Iowa | IA | 4 |
| 20 | Kansas | KS | 4 |
| 27 | Minnesota | MN | 4 |
| 29 | Missouri | MO | 4 |
| 31 | Nebraska | NE | 4 |
| 38 | North Dakota | ND | 4 |
| 46 | South Dakota | SD | 4 |
| 95 | Southeast Region | SEST | 5 |
| 1 | Alabama | AL | 5 |
| 5 | Arkansas | AR | 5 |
| 12 | Florida | FL | 5 |
| 13 | Georgia | GA | 5 |
| 21 | Kentucky | KY | 5 |
| 22 | Louisiana | LA | 5 |
| 28 | Mississippi | MS | 5 |
| 37 | North Carolina | NC | 5 |
| 45 | South Carolina | SC | 5 |
| 47 | Tennessee | TN | 5 |
| 51 | Virginia | VA | 5 |
| 54 | West Virginia | WV | 5 |
| 96 | Southwest Region | SWST | 6 |
| 4 | Arizona | AZ | 6 |
| 35 | New Mexico | NM | 6 |
| 40 | Oklahoma | OK | 6 |
| 48 | Texas | TX | 6 |
| 97 | Rocky Mountain Region | RKMT | 7 |
| 8 | Colorado | CO | 7 |
| 16 | Idaho | ID | 7 |
| 30 | Montana | MT | 7 |
| 49 | Utah | UT | 7 |
| 56 | Wyoming | WY | 7 |
| 98 | Far West Region | FWST | 8 |
| 2 | Alaska | AK | 8 |
| 6 | California | CA | 8 |
| 15 | Hawaii | HI | 8 |
| 32 | Nevada | NV | 8 |
| 41 | Oregon | OR | 8 |
| 53 | Washington | WA | 8 |

**List of Attributes**

| **Attribute** | **Description** |
| --- | --- |
| high | High ILI activity in the current week. |
| low | Low ILI activity in the current week. |
| minimal | Minimal ILI activity in the current week. |
| moderate | Moderate ILI activity in the current week. |
| has_been_stable | ILI activity has been stable. |
| has_decreased | ILI activity has decreased. |
| has_increased | ILI activity has increased. |
| will_be_stable | ILI activity will be stable in current week. |
| will_decrease | ILI activity will decrease in current week. |
| will_increase | ILI activity will increase in current week. |
| was_high | ILI activity was high before the current week. |
| was_low | ILI activity was low before the current week. |
| was_minimal | ILI activity was moderate before the current week. |
| was_moderate | ILI activity was moderate before the current week. |
| was1_high | ILI activity was high one week ago. |
| was1_low | ILI activity was low one week ago. |
| was1_minimal | ILI activity was minimal one week ago. |
| was1_moderate | ILI activity was moderate one week ago. |
| was2_high | ILI activity was high two weeks ago. |
| was2_low | ILI activity was low two weeks ago. |
| was2_minimal | ILI activity was minimal two weeks ago. |
| was2_moderate | ILI activity was moderate two weeks ago. |
| was3_high | ILI activity was high three weeks ago. |
| was3_low | ILI activity was low three weeks ago. |
| was3_minimal | ILI activity was minimal three weeks ago. |
| was3_moderate | ILI activity was moderate three weeks ago. |
| was4_high | ILI activity was high four weeks ago. |
| was4_low | ILI activity was low four weeks ago. |
| was4_minimal | ILI activity was minimal four weeks ago. |
| was4_moderate | ILI activity was moderate four weeks ago. |
| was52_high | ILI activity was high 52 weeks ago. |
| was52_low | ILI activity was low 52 weeks ago. |
| was52_minimal | ILI activity was minimal 52 weeks ago. |
| was52_moderate | ILI activity was moderate 52 weeks ago. |
| FWST | Far West Region |
| GLAK | Great Lakes Region |
| MEST | Mideast Region, |
| NENG | New England Region |
| PLNS | Plains Region |
| RKMT | Rocky Mountain Region |
| SEST | Southeast Region |
| SWST | Southwest Region |
